# Unilateral pulmonary agenesis

**DOI:** 10.1590/S1806-37132014000300017

**Published:** 2014

**Authors:** Nulma Souto Jentzsch

**Affiliations:** São José University Hospital, and Researcher, Minas Gerais School of Medical Sciences, Belo Horizonte, Brazil

To the Editor:

I read with great interest the article by Malcon et al. reporting the occurrence of unilateral pulmonary agenesis in an 8-year-old asymptomatic male child without other associated malformations, and I congratulate the authors on it.^(^ ^1^ ^)^

**Figure 1:**
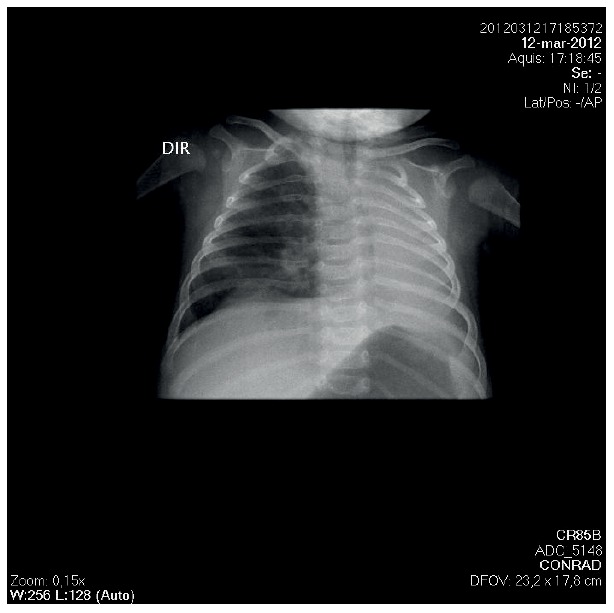
Chest X-ray showing absence of the left lung.

**Figure 2:**
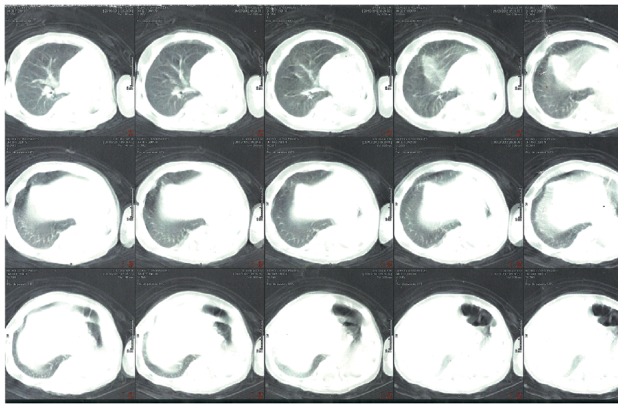
Chest CT scans showing absence of the left lung.

I would like to report that we treated a 3-month-old female infant, from the city of Belo Horizonte, Brazil, who had been born at term and had undergone prenatal testing uneventfully. The infant was taken to the Department of Pediatrics of the São José University Hospital in that same city on March of 2012 with a 4-day-history of cough and fever. Her parents reported that she had no comorbidities or previous hospitalizations. The patient presented with acute respiratory failure and required oxygen by nasal catheter. Examination of the respiratory system revealed diminished breath sounds throughout the left hemithorax, without adventitious sounds. A chest X-ray (Figure 1) showed complete opacification of the left hemithorax, together with deviation of the trachea and mediastinum to the left. The left lung was not seen on chest CT (Figure 2). Doppler echocardiography showed agenesis of the left pulmonary artery, without other cardiac abnormalities, and bronchoscopy revealed complete absence of the left lung and absence of bronchial stump. A diagnosis of left lung agenesis was therefore established. The patient's course was satisfactory, and she is under outpatient follow-up. Congenital malformations of the lung are rare and vary widely in their clinical presentation and severity, depending mostly on the degree of lung involvement and their location in the thoracic cavity.^(^ ^2^ ^)^ The earliest stage of lung development occurs during the first 50 days of gestation and is called embryonic stage: around the 26th day, the anterior part of the foregut invaginates and forms the laryngotracheal bud; subsequently, the two main bronchi are formed. After 48 days of gestation, the segmental and subsegmental bronchi start forming. The pulmonary arteries form from the sixth aortic arch, and the pulmonary veins form from the invagination of the sinoatrial region of the heart. The development of the conducting airways starts early, whereas the respiratory bronchioles, alveolar ducts, and alveoli form later in gestation, in the stages called pseudoglandular, canalicular, saccular, and alveolar.

In unilateral pulmonary agenesis, the right or left main bronchus does not develop, and there is absence of bronchi, parenchyma, and pulmonary vessels. The origin of pulmonary agenesis is unknown, and its prevalence, including the bilateral and unilateral forms, is 0.5-1.0 per 10,000 live births. The bilateral form is incompatible with life.^(^ ^3^ ^)^

In unilateral pulmonary agenesis, the mortality rate in the neonatal period is approximately 50%, especially if there are other associated malformations (especially cardiac malformations). ^(^ ^4^ ^)^ Musculoskeletal, gastrointestinal, and renal abnormalities may also be present. The mortality rate is higher when there is agenesis of the right lung. This difference can be explained by a greater mediastinal shift, leading to tracheal compression. ^(^ ^3^ ^)^ Agenesis of the left lung is more common, causing compensatory growth of the remaining lung and its herniation into the contralateral hemithorax.^(^ ^5^ ^)^

Asymptomatic patients do not require intervention, especially in the absence of associated anomalies. However, pulmonary infections or other lung diseases should be treated early, and the patient should have clinical follow-up to detect possible abnormalities, such as pulmonary hypertension. Sometimes, the diagnosis of unilateral pulmonary agenesis is delayed, being made in adulthood in asymptomatic patients. Other associated malformations and recurrent respiratory infections are factors that aid in earlier diagnosis.

The prognosis is better when there is agenesis of the left lung and when there are no cardiac malformations.
