# Supplementary material for: Unilateral pulmonary agenesis
Source: J Bras Pneumol. 2014 May-Jun;40(3):322–4. doi: 10.1590/S1806-37132014000300017 (PMC4109206; doi:10.1590/S1806-37132014000300017)
Supplement: Translation

# Translation: Agenesia pulmonar unilateral

**Authors:** Nulma Souto Jentzsch

**Affiliations:** Hospital Universitário São José, e Pesquisadora, Instituto de Pós-Graduação da Faculdade de Ciências Médicas de Minas Gerais. Belo Horizonte (MG) Brasil

Ao Editor:

Li com bastante interesse e congratulo os autores Malcon et al. que relatam a ocorrência de agenesia pulmonar unilateral em uma criança assintomática do sexo masculino de oito anos de idade, sem outras malformações associadas.^(^ ^1^ ^)^

Gostaríamos de relatar que atendemos uma criança do sexo feminino, de três meses de idade, procedente da cidade de Belo Horizonte (MG), nascida a termo, com exames pré-natais realizados sem intercorrências. Ela foi conduzida ao serviço de pediatria do Hospital Universitário São José, localizado na mesma cidade, em março de 2012, com queixa de tosse e febre que duravam 4 dias. Os pais negavam quaisquer comorbidades ou internações prévias da criança. A paciente apresentava-se com insuficiência respiratória aguda e necessitava de oxigênio por cateter nasal. Ao exame do aparelho respiratório, encontrou-se murmúrio vesicular diminuído em todo o hemitórax esquerdo, sem ruídos adventícios. Na radiografia de tórax (Figura 1), via-se opacificação total de hemitórax esquerdo, com traqueia e mediastinos desviados para a esquerda. Na TC de tórax, o pulmão esquerdo não foi visualizado (Figura 2). O ecodopplercardiograma revelou agenesia da artéria pulmonar esquerda, sem outras alterações cardíacas, e a broncoscopia demonstrou ausência completa do pulmão esquerdo e inexistência do coto brônquico. Foi estabelecido, então, o diagnóstico de agenesia pulmonar unilateral esquerda. A paciente evoluiu satisfatoriamente e está sendo acompanhada ambulatorialmente.

**Figura 1:** Radiografia de tórax revelando ausência do pulmão esquerdo.

**Figura 2:** TCs de tórax revelando ausência do pulmão esquerdo.

As malformações congênitas do pulmão são raras e variam muito na sua forma de apresentação clínica e gravidade, dependendo principalmente do grau de envolvimento pulmonar e de sua localização na cavidade torácica.^(^ ^2^ ^)^ O início do desenvolvimento pulmonar ocorre durante os 50 primeiros dias de gestação, período denominado etapa embrionária: ao redor do 26º dia, ocorre invaginação da parte anterior do intestino primitivo e se forma o broto laringotraqueal; a partir daí, são formados os dois brônquios principais. Após 48 dias de gestação se inicia a formação dos brônquios segmentares e subsegmentares. As artérias pulmonares se formam a partir do sexto arco aórtico, e as veias pulmonares formam se a partir de invaginação da parte sinoatrial do coração. O desenvolvimento das vias aéreas condutoras se inicia precocemente; já os bronquíolos respiratórios, ductos alveolares e alvéolos formam-se posteriormente na gestação, nas etapas denominadas pseudoglandular, canalicular, sacular e alveolar.

Na agenesia pulmonar unilateral, o brônquio fonte de um dos lados não se desenvolve, ocorrendo a ausência de brônquio, parênquima e vasos pulmonares. Tem origem desconhecida e sua prevalência é de 0,5-1,0 para cada 10.000 nascidos vivos, incluindo as formas bilateral e unilateral. A forma bilateral é incompatível com a vida.^(^ ^3^ ^)^

Na agenesia pulmonar unilateral, a taxa de mortalidade no período neonatal é de aproximadamente 50%, principalmente se houver outras malformações associadas, principalmente as cardíacas.^(^ ^4^ ^)^ Alterações músculo-esqueléticas, gastrointestinais e renais também podem existir. A taxa de mortalidade é maior quando a agenesia é no pulmão direito. Tal diferença pode ser explicada por um maior desvio mediastinal, levando a compressão traqueal.^(^ ^3^ ^)^ O acometimento da agenesia é mais frequente à esquerda, ocasionando crescimento compensatório do pulmão remanescente e herniação do mesmo no tórax contralateral.^(^ ^5^ ^)^

Pacientes assintomáticos não necessitam de intervenção, principalmente na ausência de anomalias associadas; contudo, infecções pulmonares ou outras doenças pulmonares devem ser tratadas precocemente, e o paciente deve ter acompanhamento clínico para detectar possíveis alterações, como a hipertensão pulmonar. Às vezes, o diagnóstico da agenesia pulmonar unilateral é mais tardio, estabelecido na vida adulta em pacientes assintomáticos. Outras malformações associadas e infecções respiratórias de repetição são fatores que auxiliam o diagnóstico mais precoce.

O prognóstico é melhor quando a agenesia pulmonar é unilateral à esquerda e quando estão ausentes malformações cardíacas.
